# Differing genetics of saline and cocaine self-administration in the hybrid mouse diversity panel

**DOI:** 10.1101/2024.12.04.626933

**Published:** 2024-12-09

**Authors:** Arshad H. Khan, Jared R. Bagley, Nathan LaPierre, Carlos Gonzalez-Figueroa, Tadeo C. Spencer, Mudra Choudhury, Xinshu Xiao, Eleazar Eskin, James D. Jentsch, Desmond J. Smith

**Affiliations:** 1 Department of Molecular and Medical Pharmacology, Geffen School of Medicine, UCLA, Los Angeles, CA 90095; 2 Department of Psychology, Binghamton University, Binghamton, NY 13902; 3 Department of Computer Science, UCLA, Los Angeles, CA 90095; 4 Department of Integrative Biology and Physiology, UCLA, Los Angeles, CA 90095; 5 Department of Computational Medicine, UCLA, Los Angeles, CA 90095; 6 Current address: Cedars-Sinai Medical Center, 8700 Beverly Blvd, Los Angeles, CA 90048; 7 Current address: Department of Pharmaceutical Sciences, Binghamton University, Binghamton, NY 13902; 8 Current address: Department of Human Genetics, University of Chicago, Chicago, IL 60637; 9 Current address: Sanford Burnham Prebys, La Jolla, CA 92037; 10 Department of Molecular and Medical Pharmacology, David Geffen School of Medicine, UCLA, Box 951735, 23-151A CHS, Los Angeles, CA 90095-1735

## Abstract

To identify genes involved in regulating the behavioral and brain transcriptomic response to the potentially addictive drug cocaine, we performed genome-wide association studies (GWASs) for intravenous self-administration of cocaine or saline (as a control) over 10 days using a panel of inbred and recombinant inbred mice. A linear mixed model increased statistical power for these longitudinal data and identified 145 loci for responding when saline only was delivered, compared to 17 for the corresponding cocaine GWAS. Only one locus overlapped. Transcriptome-wide association studies (TWASs) using RNA-Seq data from the medial frontal cortex and nucleus accumbens identified *5031434O11Rik* and *Zfp60* as significant for saline self-administration. Two other genes, *Myh4* and *Npc1*, were nominated based on proximity to loci for multiple endpoints or a *cis* locus regulating expression. All four genes have previously been implicated in locomotor activity. Our results indicate distinct genetic bases for saline and cocaine self-administration, and suggest some common genes for saline self-administration and locomotor activity.

## Introduction

In the US, over 2 million individuals use cocaine more frequently than once a month and approximately 850,000 individuals are dependent on this substance. Deaths due to cocaine overdose in 2018 were 4.5 per 100,000 standard population [1–5]. Cocaine misuse in humans has significant broad and narrow sense heritabilities of ~0.32–0.79 and ~0.27–0.30, respectively. However, identifying genes for cocaine misuse using genome-wide association studies (GWASs) in humans has been difficult because of the numerous obstacles in recruiting properly ascertained subjects.

To identify genes and loci involved in intravenous self-administration (IVSA) of cocaine, we recently used a panel of inbred and recombinant inbred mice called the hybrid mouse diversity panel (HMDP) [6,7]. As a control and to enable studies of differential gene expression associated with cocaine, a parallel experiment was performed in which saline was employed instead of cocaine, with all other factors (handling, surgery, behavioral testing) being identical. The HMDP was evaluated over 10 days using four dependent variables for cocaine and saline IVSA.

The HMDP consists of ~30 inbred and ~70 recombinant inbred mouse strains and can be used for mapping of loci involved in complex traits [8,8–10]. The inbred strains have many meiotic breakpoints, facilitating high resolution genetic mapping. The recombinant inbred strains increase statistical power. The genetic stability of the HMDP allows layering of multiple phenotypes on the panel and hence ever more powerful insights.

Though we found evidence that cocaine served as a more effective behavioral reinforcer than saline, individual strains varied considerably in the amount of saline self-administration they engaged in relative to cocaine [6,7]. Three lines of evidence indicated differing genetic causes of cocaine and saline IVSA in the HMDP. First, the behavioral endpoints were much more highly correlated within than between infusates. Second, both narrow and broad sense heritabilities were significantly higher for saline IVSA (~0.31 and ~0.44, respectively) than cocaine (~0.20 and ~0.32, respectively). Third, the genome scans for cocaine and saline using individual days segregated nearly completely when subjected to unsupervised clustering, although neither scan had significant loci.

To increase the statistical power of the cocaine GWASs, we took advantage of the longitudinal data using a linear mixed model implemented in GMMAT software. The model employed fixed and random effects of testing day as a continuous variable and corrected for population structure using a genetic relatedness matrix. A total of 15 unique significant cocaine loci were identified. To further increase statistical power, we also used transcriptome-wide association studies (TWASs) to combine the longitudinal genome scans with RNA-Seq data from medial frontal cortex (mFC) and nucleus accumbens (NAc) of the cocaine cohort.

The TWASs identified 17 additional genes for the cocaine IVSA endpoints. Both the TWASs and GWASs highlighted the *Trpv2* ion channel as a key locus for cocaine self-administration. *Trpv2* is activated upon cannabinoid binding, allowing calcium ions to enter neurons, and is classified as an ionotropic cannabinoid receptor. The identification of *Trpv2* as a gene for cocaine IVSA may partly explain the common genetic risk factors for cocaine addiction and cannabis use [11]. Increased expression of *Trpv2* was associated with decreased cocaine IVSA in HMDP, suggesting that this ion channel may be an attractive target for development of therapeutics for cocaine use disorder.

In this report, we describe longitudinal GWASs of the saline IVSA dataset in the HMDP. We also employ TWASs to identify additional genes for saline IVSA using RNA-Seq data from this cohort.

## Materials and Methods

### Saline and cocaine intravenous-self administration

A total of 479 and 477 mice from 84 strains of the HMDP were used for cocaine and saline IVSA respectively, as described [6,7]. Animals were tested for cocaine or saline IVSA over 10 consecutive daily sessions. Testing chambers had two response levers, one of which, when actuated, produced an infusion of cocaine or saline depending on cohort, the other of which was inactive. To encourage conditioning, infusion of the agent was signaled by a visual cue (flashing of the house light) for 20 s. Pressing the inactive lever had no programmed effect. After an infusion, active lever presses were recorded but no infusion given for 20 s. The testing was 2 h or until 65 infusions were given, whichever came first.

### Mapping loci for IVSA

Loci for saline IVSA were mapped using GMMAT to evaluate the normalized endpoints as longitudinal traits over the 10 testing days, as described [7,12]. This linear mixed model included fixed and random effects of testing day as well as correcting for population structure via a kinship matrix. Covariates were sex, active lever (left or right), testing chamber, cohort and age. Genome-wide significance thresholds of 5% obtained from permutation were employed. Genotypes using single nucleotide polymorphism (SNPs) were obtained [13], and after removing SNPs with minor allele frequency < 5% or missing genotype frequency > 10%, 340,097 SNPs remained. Mouse genome build GRCm38/mm10 was employed for coordinates [14].

### Open field

Comparison of saline IVSA endpoints and open field locomotor activity used data downloaded from the Mouse Phenome Database [15]. The open field measures were normalized distance traveled in the first 10 min of a 55 min testing session for 62 inbred strains [16] and a 20 min testing session of 55 BXD recombinant inbred strains [17].

### RNA-Seq

RNA-Seq was performed on NAc (core and shell) and mFC of 41 strains exposed to either the cocaine or saline IVSA [6,7]. A total of 73 M reads of 75 bp paired-ends were obtained per region per strain. GWASs of transcript, spliceform and RNA editing abundance were performed as described using FaST-LMM to correct for population structure [6,7,18,19]. Covariates were sex and sequencing batch. *Cis* expression quantitative trait loci (eQTLs), splicing (percent spliced in, or Ψ) QTLs (ΨQTLs) and editing QTLs (ϕQTLs) were defined as residing within 2 Mb of the regulated gene. Behavioral or molecular QTLs, were deemed coincident if located < 2 Mb apart [18]. The ascertainment rate for RNA editing was defined as the proportion of informative sequence reads for a given site. All editing sites were A to I.

### Transcriptome-wide association studies

FUSION and FOCUS software were used to perform transcriptome-wide association studies (TWASs) [20,21]. FUSION significance thresholds used *P* < 0.05, Bonferroni corrected for the number of genes tested. FOCUS significance thresholds employed a posterior inclusion probability (pip) > 0.8.

### eCAVIAR

eCAVIAR was used to find single nucleotide polymorphisms (SNPs) that co-regulated *cis* eQTLs and behavioral loci with the highest colocalization posterior probability (CLPP) [22]. Markers within 200 SNPs of the *cis* eQTL were evaluated. The support threshold for a co-regulating SNP was CLPP > 0.01.

### Data availability

The sequencing data generated in this study can be downloaded from the NCBI BioProject database (https://www.ncbi.nlm.nih.gov/bioproject/) under accession number PRJNA755328. Data and code are also available from figshare (https://figshare.com/; doi: 10.6084/m9.figshare.27958836).

## Results

### Saline self-administration

We evaluated 84 strains of the HMDP for IVSA of saline (477 mice) or cocaine (479 mice) over a 10 day testing period, as described previously [6]. Pressing one lever in the testing chamber caused delivery of the infusate (saline or cocaine, depending on experiment), while the other lever was inactive. Infusate delivery was accompanied by flashing of the house light. Four behavioral endpoints were evaluated: number of infusions, active lever presses, percent active lever presses and inactive lever presses.

Genome-wide association studies (GWASs) for saline IVSA using the four endpoints on each of the ten days produced no genome-wide significant loci [6,7]. To increase statistical power, we used GMMAT software to incorporate the longitudinal phenotypes via a linear mixed model [12]. The day of assay was used as both a fixed and random effect together with random effects of SNPs to correct for genetic relatedness, as described [6,7].

**Figure 1.**
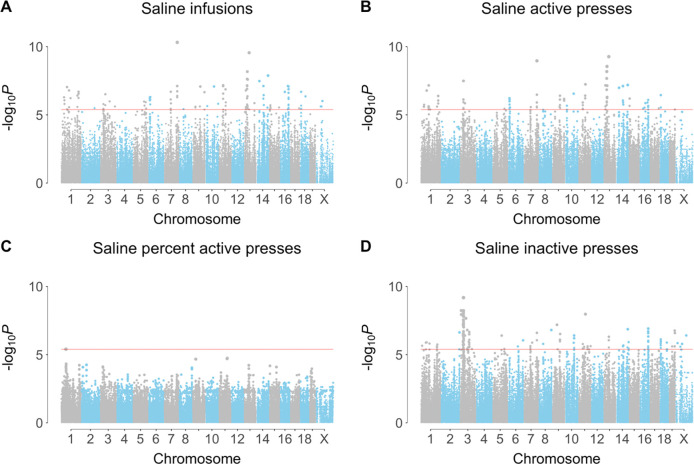
Longitudinal genome scans for saline IVSA. (**A**) Infusions. (**B**) Active lever presses. (**C**) Percent active lever presses, (**D**) Inactive lever presses. Red horizontal line, family-wise error rate = 5%.

A total of 145 significant loci were identified for saline IVSA using the four behavioral endpoints, of which 85 were unique (Figure 1, Table S1, Figure S1). Of the 145 loci for saline IVSA, 56 were for infusions, 35 were for active lever presses, 1 for percent active lever presses and 53 for inactive lever presses. In contrast, 17 significant loci were obtained for cocaine using GMMAT, of which 15 were unique [7]. The greater number of loci for saline IVSA is consistent with the higher broad and narrow sense heritabilities of this infusate.

The only locus significant for both cocaine and saline IVSA was for inactive lever presses on Chromosome 3 [7]. The peak SNP for saline was rs30114031 (37,799,968 bp, *P* = 6.6 × 10^−10^), which was more significant than the peak SNP for cocaine, rs30059671 (38,178,200 bp, *P* = 3.2 × 10^−7^). *Spry1* is centromeric to these SNPs, but closer to the saline peak (157,696 bp distant) than the cocaine peak (535,928 bp).

**Figure 2.**
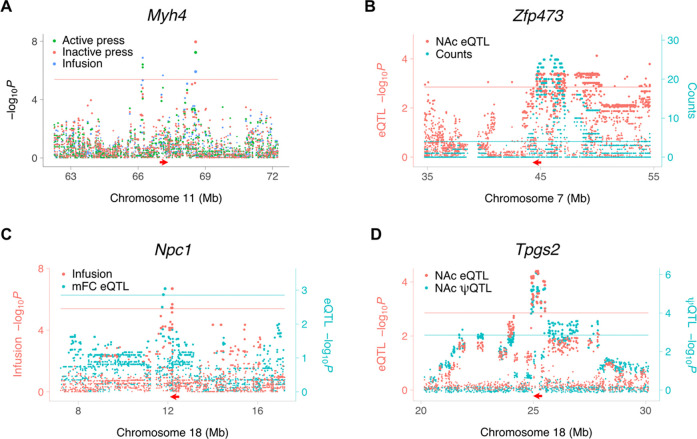
Regulation of gene expression in NAc and mFC of saline-exposed mice. (**A**) Myh4 is close to loci for three different saline IVSA endpoints. (**B**) Co-aligned NAc eQTL hotspot and Zfp473 cis eQTL. Red arrow, location of Zfp473. Blue horizontal line, eQTL hotspot significance threshold, FDR < 0.05. Red horizontal line, cis eQTL significance threshold. (**C**) Coincident loci for saline infusions and Npc1 cis eQTL in mFC. (**D**) Coincident NAc cis ΨQTL for exon 6 and eQTL for Tpgs2. Peak marker rs31436205 for both QTLs. Blue and red horizontal lines, respective significance thresholds.

A triplet of loci for infusions, active lever presses, and inactive lever presses spanned from 66,206,986 bp to 68,567,223 bp (2,360,237 bp) on Chromosome 11 (Figure 2A). *Myh4* was located at the approximate center of this region (67,249,238 bp) and encodes a myosin heavy chain gene. Selection of mice for high voluntary wheel running resulted in mini-muscle mice, which have decreased muscle mass as a result of an intronic SNP in *Myh4* [23].

### RNA-Seq

To identify pathways for saline IVSA, RNA-Seq was performed on NAc and mFC from 41 of the cocaine- and saline-exposed strains from the HMDP. NAc and mFC were chosen because of their role in operant self-administration of cocaine [2,5]. A total of ~ 73 M paired-end reads were obtained for each brain region per strain [6]. Transcripts, splicesomes and RNA editing sites regulated by infusate, brain region and sex were previously discussed [7].

### Expression QTLs

*Cis* and *trans* QTLs were mapped for transcript abundance (expression QTLs, or eQTLs), splicing (sQTLs or ΨQTLs) and RNA editing (edit QTLs or ϕQTLs) in the saline samples using FaST-LMM [19]. The number of *cis* eQTLs averaged over the two brain regions for saline was 4,878 ± 253. The distance between the *cis* eQTLs and their corresponding gene was 0.62 Mb ± 0.009 Mb, averaged across brain regions, and is consistent with the linkage disequilibrium of the HMDP [8,9,24].

Hotspots, in which a locus regulates many genes, were identified for transcript abundance [10,18]. A total of 12 hotspots regulating ≥ 20 genes were present in NAc saline samples (FDR < 2.2 × 10^−16^), and 22 hotspots in mFC saline samples (FDR < 2.2 × 10^−16^). We sought candidate genes for hotspots by looking for co-aligned *cis* eQTLs. One NAc saline hotspot was coincident with a *cis* eQTL for the transcription factor *Zfp473* (Figure 2B).

*Npc1* showed a *cis* eQTL co-aligned with a QTL for infusions, suggesting a possible link between *Npc1* and the behavioral phenotype (Figure 2C). This gene was significant in a human GWAS study for walking pace (*P* = 3 × 10^−11^) [25,26].

### Splicing QTLs

A total of 1,418 ± 46 *cis* splicing QTLs (ΨQTLs) were detected for saline exposed mice, averaged over the two brain regions. Transcript abundance can be affected by genetic variants that alter spliceform preference and hence mRNA stability [27,28]. To evaluate the prevalence of this phenomenon, we examined whether there was a statistically significant enrichment in coincident *cis* eQTLs and ΨQTLs. There were significant enrichments in both NAc and mFC. A total of 362 coincident *cis* eQTLs and ΨQTLs were found in NAc from saline treated mice (odds ratio, OR, = 2.7, *P* < 2.2 × 10^−16^, Fisher’s exact test), and 398 in mFC (OR = 2.6, *P* < 2.2 × 10^−16^). Similar results were found for the cocaine exposed samples [7], indicating this enrichment is not infusate specific.

**Figure 3.**
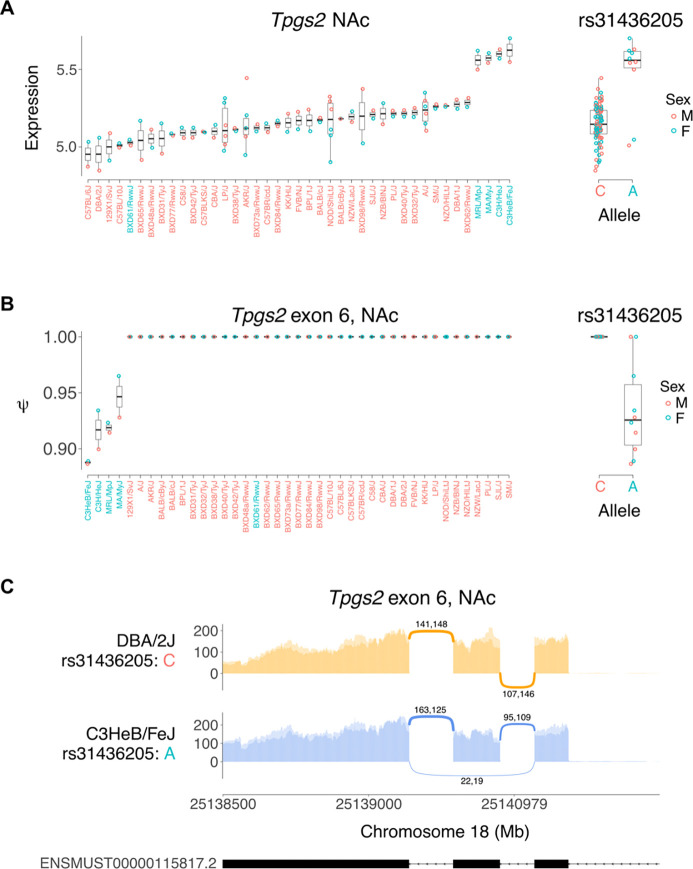
Genetic variation in alternate splicing of exon 6 Tpgs2 in saline treated mice. (**A**) Strain survey of Tpgs2 expression in saline NAc. Peak eQTL for Tpgs2 is rs31436205. Strains with C allele have low expression (red), A allele have high expression (blue). (**B**) Percent spliced in (psi or Ψ) for exon 6 of Tpgs2. Strains with C allele have close to 100% Ψ, while strains with A allele have lower Ψ. (**C**) Sashimi plot [29] showing high Ψ for Tpgs2 exon 6 in DBA2/J, a strain with C allele of rs31436205, and low Ψ for C3HeB/FeJ, a strain with A allele.

An example of a coincident *cis* eQTL and *cis* ΨQTL for exon 6 of *Tpgs2* in NAc of saline exposed mice is shown in Figure 2D. The peak SNP, rs31436205, was the same for both the *cis* eQTL and *cis* ΨQTL (Figure 3). The C allele was associated with higher percent spliced in of exon 6 of *Tpgs2* and with lower transcript abundance, consistent with inclusion of this exon destabilizing the mRNA.

### Editing QTLs

Sequence changes caused by RNA editing can alter transcript stability and abundance as well as changing coding sequence [30]. A total of 262 ± 31 *cis*-acting loci were identified that affect RNA editing efficiency (ϕQTLs) in saline-exposed mice averaged over the two brain regions [31]. Convincing quantitation of RNA editing event is more demanding than for transcript or spliceform abundance, since the editing occurs at single nucleotides. Editing events showed an ascertainment rate of 37 ± 0.5% across RNA-Seq samples from saline-exposed mice averaged over the two brain regions. The less than 100% detection rate implies decreased statistical power and indicates that the ϕQTLs should be treated with some caution.

We looked for statistically significant enrichment in coincident *cis* eQTLs and ϕQTLs to evaluate how often genetically determined variations in RNA editing can change transcript levels. In NAc from saline treated mice, there were 57 co-aligned *cis* eQTLs and ϕQTLs (odds ratio, OR, = 2.1, *P* = 1.8 × 10^−6^) and 45 in mFC (OR = 1.8, *P* = 4.7 × 10^−4^, Fisher’s exact test), both significant enrichments. Co-aligned *cis* ϕQTLs and *cis* eQTLs should show significantly increased numbers of editing sites located in the mature mRNA compared to intronic or intergenic regions, assuming that *cis* ϕQTLs regulate editing events which in turn alter transcript stability and result in *cis* eQTLs. Indeed, we found significantly increased numbers of editing sites in 5’ untranslated, 3’ untranslated and exonic coding regions compared to intronic and intergenic regions for the coincident *cis* ϕQTLs and eQTLs in both NAc (odds ratio = 2.0, *P* = 3.2 × 10^−3^, Fisher’s Exact Test) and mFC (odds ratio = 2.5, *P* = 2.0 × 10^−4^, Fisher’s Exact Test) of saline treated mice.

Statistical enrichment of coincident *cis* eQTLs and ϕQTLs, as well as a corresponding enrichment of editing sties in 5’ untranslated, 3’ untranslated and exonic coding regions was also found in cocaine exposed mice [7]. The replication of these findings in saline treated mice indicates that these phenomenon are not infusate specific.

### Transcriptome-wide association studies

To pinpoint individual genes for saline IVSA, we employed transcriptome-wide association studies (TWASs). This approach acknowledges that the bulk of complex trait loci act through variation in expression regulatory sequences, and hence transcript levels, rather than amino acid sequence changes [32]. A gene for a trait is identified by TWAS when the genetically-predicted part of the gene’s expression is significantly correlated with the trait. TWAS enhances statistical power because the approach analyzes loci at the gene rather than marker level, leading to decreased multiple hypothesis correction. FUSION and FOCUS packages were employed to perform TWAS, with FOCUS providing additional fine mapping compared to FUSION [20,21].

**Figure 4.**
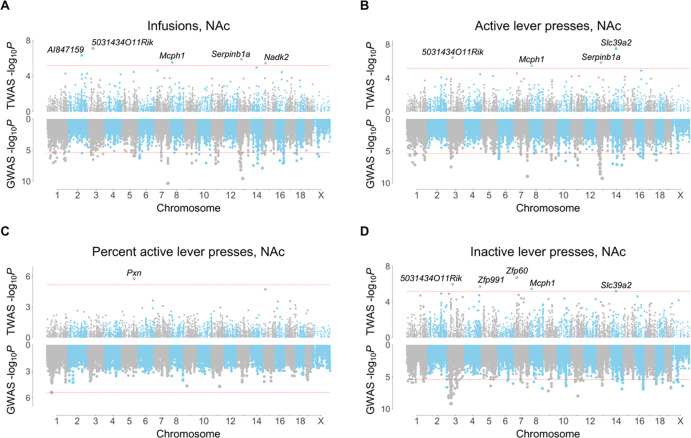
FUSION TWAS for saline IVSA using NAc RNA-Seq. (**A**) Infusions. (**B**) Active lever presses. (**C**) Percent active lever presses. (**D**) Inactive lever presses. TWASs on top, GWASs on bottom.

A total of 15 genes were uncovered using FUSION of saline exposed NAc, of which 9 were unique (Figure 4). Five genes were uncovered using saline exposed mFC, of which 4 were unique (Figure S2). There was no overlap between the NAc and mFC TWASs. A total of 5 unique genes were significant using FOCUS in NAc (active press: *Serpinb1a*, pip = 0.97; *Pcnp*, pip = 0.91; inactive press: *Cbx2*, pip = 0.94) and in mFC (infusions: *5031434O11Rik*, pip = 0.95; active press: *5031434O11Rik*, pip = 0.90; inactive press: *Tmem241*, pip = 0.94). Two genes, *5031434O11Rik* and *Serpinb1a*, were significant using both FUSION and FOCUS. We found no significant associations for phenotypes related to locomotor activity in a human GWAS catalog using genes identified in the TWASs [25]. FUSION identified *Zfp60*, a zinc finger gene, as significant for inactive lever presses in NAc of saline exposed mice (Figure 4). Decreased *Zfp60* expression was associated with increased inactive lever presses. This gene is expressed in olfactory bulb, hippocampus, cortical subplate and hypothalamus [33]. Consistent with the TWAS finding, knockouts of *Zfp60* show significant hyperactivity based on multiple endpoints of both the open-field test and light-dark test (*P* = 7.9 × 10^−7^ and 4.5 × 10^−5^, respectively) [34].

**Figure 5.**
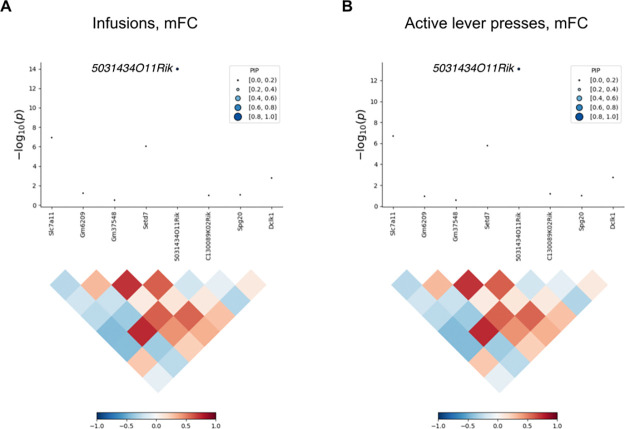
FOCUS of mFC in saline exposed mice. (**A**) FOCUS of 5031434O11Rik for infusions. Linkage disequilibrium map shown underneath. (**D**) FOCUS of 5031434O11Rik for active lever presses.

Another gene, *5031434O11Rik*, was significant for three out of four IVSA endpoints (infusions, active lever presses and inactive lever presses) using FUSION of saline exposed NAc (Figure 4). The gene was also significant for infusions (pip = 0.95, −log_10_*P* = 14) and active lever presses (pip = 0.90, −log_10_*P* = 13) using FOCUS of saline exposed mFC (Figure 5). Increased expression of *5031434O11Rik* was associated with increased lever presses.

**Figure 6.**
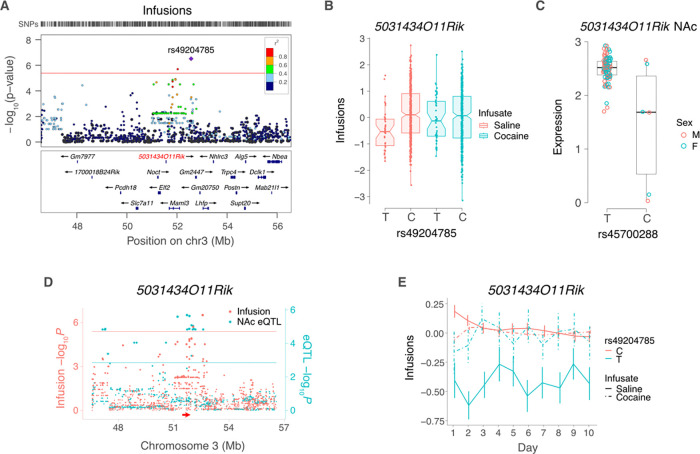
5031434O11Rik and saline infusions. (**A**) Locuszoom plot [35] for saline IVSA infusions, showing locus harboring 5031434O11Rik (red). Peak SNP is rs49204785. R^2^ values indicate linkage disequilibrium. (**B**) The peak behavior SNP shows significant allelic effect for saline but not cocaine infusions. (**C**) Expression of 5031434O11Rik in saline NAc. Peak eQTL for 5031434O11Rik is rs45700288. (**D**) Coincident loci for saline infusions and 5031434O11Rik cis eQTL in saline NAc. (**E**) Infusion time course for peak behavior SNP of 5031434O11Rik locus, rs49204785. Means ± s.e.m.

The peak SNP for infusions near to *5031434O11Rik* on Chromosome 3 was rs49204785 at 52,563,394 bp, located 999,818 bp telomeric to the gene (Figure 6A). The C allele of rs49204785 was associated with significantly higher saline infusions compared to the T allele (effect size = 0.40 ± 0.08, *P* = 3.1 × 10^−7^) (Figure 6B). Consistent with the effect of *5031434O11Rik* being saline specific, there was no significant allelic difference of rs49204785 for cocaine.

The peak SNP for the *5031434O11Rik cis* eQTL was rs45700288 (51,711,018 bp), which was in significant linkage disequilibrium with rs49204785 (D′ = 0.83, *R*^2^ = 0.63, *P* < 2.2 × 10^−16^) (Figures 6C,D). The T allele of rs49204785 showed significantly decreased saline infusions (linear mixed model: fixed effect, day of test; random intercept of strain; interaction between SNP and infusate: Z = 6.2, *P* = 6.8 × 10^−10^) (Figure 6E). In contrast, the effects of the C allele on saline infusions and either allele on cocaine infusions were not significantly different (linear mixed model: fixed effect, day of test; random intercept of strain; Z < 1.3, *P* > 0.41).

### eCAVIAR

We used eCAVIAR to further evaluate the contribution of *5031434O11Rik* to saline IVSA. eCAVIAR accounts for the uncertainty introduced by linkage disequilibrium while evaluating the posterior probability that both GWAS and eQTLs are caused by the same SNP [22]. Support for the same causal variant is given by a colocalization posterior probability (CLPP) > 0.01.

Consistent with a causal role for saline IVSA suggested by the TWASs, eCAVIAR identified the peak SNP for the *5031434O11Rik cis* eQTL and the behavioral endpoints as rs48952099 (52,050,163 bp on Chromosome 3; infusions, NAc CLPP = 0.016; mFC CLPP = 0.018; active lever presses, NAc CLPP = 0.015; mFC CLPP = 0.016). This SNP was roughly half-way between the peak eQTL SNP (rs45700288; 51,711,018 bp) and the peak SNP for saline infusions (rs49204785; 52,563,394 bp).

### 5031434O11Rik

The *5031434O11Rik* gene encodes a long non-coding (lnc) RNA gene and is expressed in the olfactory region, cortex, hippocampus and cerebellum [33]. A recent study found that *5031434O11Rik* was the most strongly differentially expressed gene in the striatum of four mouse lines selectively bred for high voluntary wheel running compared to non-selected controls [36]. Increased expression of *5031434O11Rik* was associated with decreased activity, opposite to our TWAS results. The different genetic backgrounds in the wheel running and present studies may explain the contrasting relationship.

The authors of the wheel running study remarked that *5031434O11Rik* overlaps with, and is a potential antisense transcript to, the 5′ end of a neighboring gene, *Setd7*. Since *Setd7* is a histone lysine methyltransferase, *5031434O11Rik* may act by degrading the *Setd7* transcript and altering chromatin. However, we found no significant relationship between *5031434O11Rik* and *Setd7* transcript levels in the combined saline NAc and mFC samples analyzed using a linear mixed model that used tissue and strain as random effects (t[1,108] = 1.5, *P* = 0.14). Similarly, there was no significant correlation between *5031434O11Rik* and *Setd7* transcript levels in an RNA-Seq atlas of mouse tissues using tissue as a random effect (t[1,32] = 1.9, *P* = 0.07) [37]. These observations suggest that *5031434O11Rik* may regulate locomotor activity by a mechanism independent of chromatin or, if the gene does modify chromatin, it does not act by changing *Setd7* transcript levels.

### Saline IVSA and open field

The endpoint of percent active lever presses was our attempt to normalize IVSA based on locomotor activity. Of the 145 unique behavioral loci for saline IVSA, only one was significant for percent active lever presses. In addition, only one gene was significant for percent lever presses using FUSION, FOCUS and eCAVIAR. These observations suggest that the loci for the other saline IVSA endpoints may be detecting genetic effects for phenotypes related to spontaneous locomotor activity. Contrary to this conclusion, there was no significant relationship between the saline IVSA endpoints and distance traveled in the open field for combined data from inbred and BXD recombinant inbred strains (*R* < 0.12, *P* > 0.34) [15–17].

## Discussion

Our previous studies employed longitudinal linear mixed models to identify 17 behavioral loci for cocaine IVSA in the HMDP [6,7]. In this work, we used the same approach to analyze a parallel group of control mice that had been subjected to saline IVSA. We found 145 loci for the saline IVSA, only one of which overlapped with the cocaine loci. The much larger number of loci for saline compared to cocaine is consistent with the higher heritabilities of saline (both broad and narrow sense) than cocaine. These observations together suggest a differing and simpler genetic architecture for saline compared to cocaine. Studies of greater statistical power may begin to reveal additional loci for cocaine IVSA.

One region on Chromosome 11 harbored three loci that regulated saline infusions, active lever presses and inactive lever presses. In the middle of this region was *Myh4*, a myosin heavy chain gene. In a previous study, an intronic SNP in *Myh4* was positively selected in mouse lines bred for voluntary wheel running. This SNP causes the mini-muscle phenotype, which results in decreased muscle mass and faster but decreased duration of wheel running. These observations suggest that genetic regulation of saline IVSA can be due to non-neural as well as neural effects.

Significant enrichment of ΨQTLs and ϕQTLs co-aligned with their cognate *cis* eQTLs was found in both saline exposed NAc and mFC. In addition, coincident *cis* ϕQTLs and eQTLs showed significant enrichment of editing sites in 5’ and 3’ untranslated regions and also exonic coding regions compared to intronic or intergenic regions. Splicing or RNA editing can thus alter transcript abundance by changing mRNA stability. Similar results were found in NAc and mFC from mice that underwent cocaine IVSA, suggesting that this phenomenon is general. *Npc1* possessed a *cis* eQTL that was coincident with a QTL for saline infusions, suggesting a possible role for the gene in this behavior. *Npc1* was also significant in a human GWAS study for walking pace

A total of 13 unique genes were implicated in saline IVSA using the FUSION TWASs. FOCUS TWASs identified a total of 5 unique genes, of which two overlapped with the FUSION results. The lnc RNA gene, *5031434O11Rik*, in particular, was implicated in saline infusions, active lever presses and inactive lever presses using FUSION TWAS, and in infusions and active lever presses using FOCUS TWAS and eCAVIAR. *5031434O11Rik* was the most strongly differentially expressed gene in lines of mice selectively bred for wheel running. Another gene emerging from the TWASs, the zinc finger gene, *Zfp60*, was found to regulate locomotor activity in a human GWAS.

Compared to protein coding genes, our understanding of lnc RNA genes is relatively poor, although an important role in brain development is becoming apparent [38,39]. Many lnc RNA genes are involved in regulation of chromatin, supporting the notion that *5031434O11Rik* may act by acting as an antisense transcript to destabilize the overlapping mRNA of its neighboring gene, *Setd7* [36]. However, we found no significant relationship between *5031434O11Rik* and *Setd7* transcript levels, suggesting that if *5031434O11Rik* acts by regulating chromatin it uses a mechanism other than destabilizing *Setd7* transcripts.

Four potential genes emerging from the saline IVSA analyses, *Myh4*, *Npc1*, *Zfp60* and *5031434O11Rik*, were implicated in locomotor activity by previous studies. Consistent with the idea that saline IVSA may be a surrogate measure of locomotion, of the 145 behavioral loci for saline IVSA, only one was significant for percent active lever presses. In addition, only one gene was significant for percent lever presses using FUSION, FOCUS and eCAVIAR. Since percent lever presses are a measure of saline IVSA normalized to locomotor activity, the relative lack of loci for this endpoint are consistent with the other endpoints being measures of locomotion. Arguing against this conclusion, there was no significant relationship between saline IVSA and distance traveled in the open field. The relative lack of significant loci for percent active lever presses also suggests that the *P* value threshold is appropriate for genome-wide association, and that the loci for the other three endpoints are unlikely to be due to noise.

Our study of saline was designed as a control for cocaine IVSA and to dissect the genetic similarities and differences between the behaviors. Indeed our results indicate distinct genetic foundations for the two traits. However, the physiological relevance of saline IVSA remains unknown. Operant responding reinforced only by a visual stimulus was correlated with subsequent lever pressing for cocaine in outbred rats that successfully acquired cocaine self-administration [40]. Nevertheless, our results suggest that reinforcement provided by the saline infusion and flashing of the house light is regulated by partially different genetic pathways than the reinforcement produced by cocaine. Notably, pressing the inactive lever does not flash the house light, and the large number of loci for inactive lever presses, some in common with infusions and active lever presses, suggests that the flashing is not the only motivating factor contributing to to active lever pressing in saline IVSA. The notion that saline IVSA is related to locomotor activity is clouded by contradictory evidence in our study. Exactly what is being measured by saline IVSA remains unclear.

## Supplementary Material

Supplement 1

Supplement 2
