# Supplementary material for: Differing genetics of saline and cocaine self-administration in the hybrid mouse diversity panel
Source: bioRxiv. 2025 Mar 29:2024.12.04.626933. Originally published 2024 Dec 9. Preprint. [Version 2] doi: 10.1101/2024.12.04.626933 (PMC11661131; doi:10.1101/2024.12.04.626933)
Supplement: Supplement 1 [file media-1.pdf]

Supplementary information: Differing genetics of saline and cocaine self-administration in the hybrid mouse diversity panel

Arshad H. Khan[1,6], Jared R. Bagley[2,7], Nathan LaPierre[3,8], Carlos Gonzalez-Figueroa[4], Tadeo C. Spencer[4], Mudra Choudhury[4,9], Xinshu Xiao[4], Eleazar Eskin[5], James D. Jentsch[2], Desmond J. Smith[1,10]

[1] Department of Molecular and Medical Pharmacology, David Geffen School of Medicine, UCLA, Los Angeles, CA 90095

[2] Department of Psychology, Binghamton University, Binghamton, NY 13902

[3] Department of Computer Science, UCLA, Los Angeles, CA 90095

[4] Department of Integrative Biology and Physiology, UCLA, Los Angeles, CA 90095

[5] Department of Computational Medicine, UCLA, Los Angeles, CA 90095

[6] Current address: Cedars-Sinai Medical Center, 8700 Beverly Blvd, Los Angeles, CA 90048

[7] Current address: Department of Pharmaceutical Sciences, Binghamton University, Binghamton, NY 13902

[8] Current address: Department of Human Genetics, University of Chicago, Chicago, IL 60637

[9] Current address: Sanford Burnham Prebys, La Jolla, CA 92037

[10] To whom correspondence should be addressed:
Department of Molecular and Medical Pharmacology
David Geffen School of Medicine, UCLA
Box 951735, 23-151A CHS
Los Angeles, CA 90095-1735
Email: DSmith@mednet.ucla.edu

Table S1. Intravenous self-administration sample sizes

Strain	Saline			Cocaine			RNA-Seq
	M	F	Total	M	F	Total	
129S1/SvImJ	3	3	6	3	3	6	None
129X1/SvJ	3	3	6	3	3	6	Same sex pooled
A/J	3	3	6	3	3	6	Individual
AKR/J	3	3	6	3	3	6	Individual
BALB/cByJ	3	3	6	3	3	6	Same sex pooled
BALB/cJ	4	3	7	4	3	7	Same sex pooled
BPL/1J	3	3	6	3	3	6	Same sex pooled
BTBR T<+> Itpr3<tf>/J	3	3	6	3	4	7	None
C3H/HeJ	3	3	6	3	3	6	Same sex pooled
C3HeB/FeJ	3	3	6	3	3	6	Same sex pooled
C57BL/10J	3	4	7	3	3	6	Same sex pooled
C57BL/6J	3	3	6	4	3	7	Same sex pooled
C57BLKS/J	3	3	6	3	3	6	Same sex pooled
C57BR/cdJ	3	3	6	3	3	6	Same sex pooled
C57L/J	5	3	8	3	3	6	None
C58/J	3	3	6	3	3	6	Same sex pooled
CBA/J	4	3	7	3	3	6	Same sex pooled
DBA/1J	3	3	6	3	3	6	Same sex pooled
DBA/2J	3	3	6	3	3	6	Same sex pooled
FVB/NJ	3	3	6	3	3	6	Same sex pooled
I/LnJ	2	2	4	1	2	3	None
KK/HlJ	3	3	6	3	3	6	Same sex pooled
LP/J	3	3	6	3	3	6	Individual
MA/MyJ	3	3	6	3	3	6	Same sex pooled
MRL/MpJ	3	3	6	4	3	7	Same sex pooled
NOD/ShiLtJ	4	3	7	3	3	6	Individual
NZB/BlNJ	2	3	5	4	3	7	Same sex pooled
NZO/HlLtJ	4	3	7	3	2	5	Same sex pooled
NZW/LacJ	3	3	6	3	3	6	Same sex pooled
PL/J	3	3	6	3	3	6	Same sex pooled
SJL/J	3	3	6	3	4	7	Same sex pooled
SM/J	3	3	6	3	4	7	Same sex pooled
BXD1/TyJ	4	3	7	3	3	6	None
BXD2/TyJ	2	3	5	3	3	6	None
BXD6/TyJ	2	1	3	2	2	4	None
BXD9/TyJ	3	3	6	3	3	6	None
BXD11/TyJ	2	3	5	3	3	6	None
BXD13/TyJ	2	3	5	3	3	6	None
BXD14/TyJ	3	2	5	2	1	3	None
BXD15/TyJ	3	3	6	2	3	5	None
BXD16/TyJ	3	3	6	3	2	5	None
BXD18/TyJ	3	2	5	2	2	4	None
BXD19/TyJ	3	3	6	3	3	6	None
BXD21/TyJ	3	1	4	3	3	6	None
BXD27/TyJ	3	2	5	3	2	5	None
BXD28/TyJ	3	3	6	2	3	5	None
BXD29/TyJ	2	2	4	2	3	5	None
BXD31/TyJ	3	3	6	4	3	7	Same sex pooled
BXD32/TyJ	3	3	6	3	3	6	Same sex pooled
BXD33/TyJ	2	3	5	1	3	4	None
BXD34/TyJ	3	4	7	3	4	7	None
BXD38/TyJ	3	3	6	3	3	6	Same sex pooled
BXD39/TyJ	3	3	6	3	3	6	None
BXD40/TyJ	3	3	6	3	2	5	Same sex pooled
BXD42/TyJ	3	3	6	3	3	6	Same sex pooled
BXD43/RwwJ	3	3	6	3	3	6	None
BXD48a/RwwJ	3	3	6	3	3	6	Same sex pooled
BXD49/RwwJ	1	2	3	3	2	5	None
BXD50/RwwJ	3	3	6	3	3	6	None
BXD51/RwwJ	3	2	5	2	2	4	None
BXD55/RwwJ	3	3	6	2	3	5	None
BXD56/RwwJ	2	3	5	2	3	5	None
BXD60/RwwJ	4	3	7	3	3	6	None
BXD61/RwwJ	3	3	6	3	3	6	Same sex pooled
BXD62/RwwJ	3	3	6	3	3	6	Same sex pooled
BXD63/RwwJ	3	3	6	2	3	5	None
BXD65/RwwJ	3	3	6	3	3	6	Same sex pooled
BXD68/RwwJ	3	3	6	3	3	6	None
BXD69/RwwJ	1	3	4	2	4	6	None
BXD70/RwwJ	3	3	6	3	3	6	None
BXD71/RwwJ	2	3	5	3	3	6	None
BXD73a/RwwJ	3	3	6	3	3	6	Same sex pooled
BXD75/RwwJ	3	2	5	2	3	5	None
BXD77/RwwJ	3	3	6	3	3	6	Same sex pooled
BXD83/RwwJ	1	3	4	3	3	6	None
BXD84/RwwJ	3	3	6	3	3	6	Same sex pooled
BXD85/RwwJ	2	3	5	3	3	6	None
BXD86/RwwJ	3	3	6	3	3	6	None
BXD89/RwwJ	3	1	4	3	1	4	None
BXD90/RwwJ	3	2	5	3	3	6	None
BXD98/RwwJ	3	3	6	3	3	6	Same sex pooled
BXD99/RwwJ	1	3	4	2	2	4	None
BXD100/RwwJ	3	3	6	3	3	6	None
BXD102/RwwJ	3	0	3	2	0	2	None
**Mean ± s.e.m.**	**2.9 ± 0.07**	**2.8 ± 0.06**	**5.7 ± 0.10**	**2.9 ± 0.06**	**2.9 ± 0.07**	**5.7 ± 0.10**	

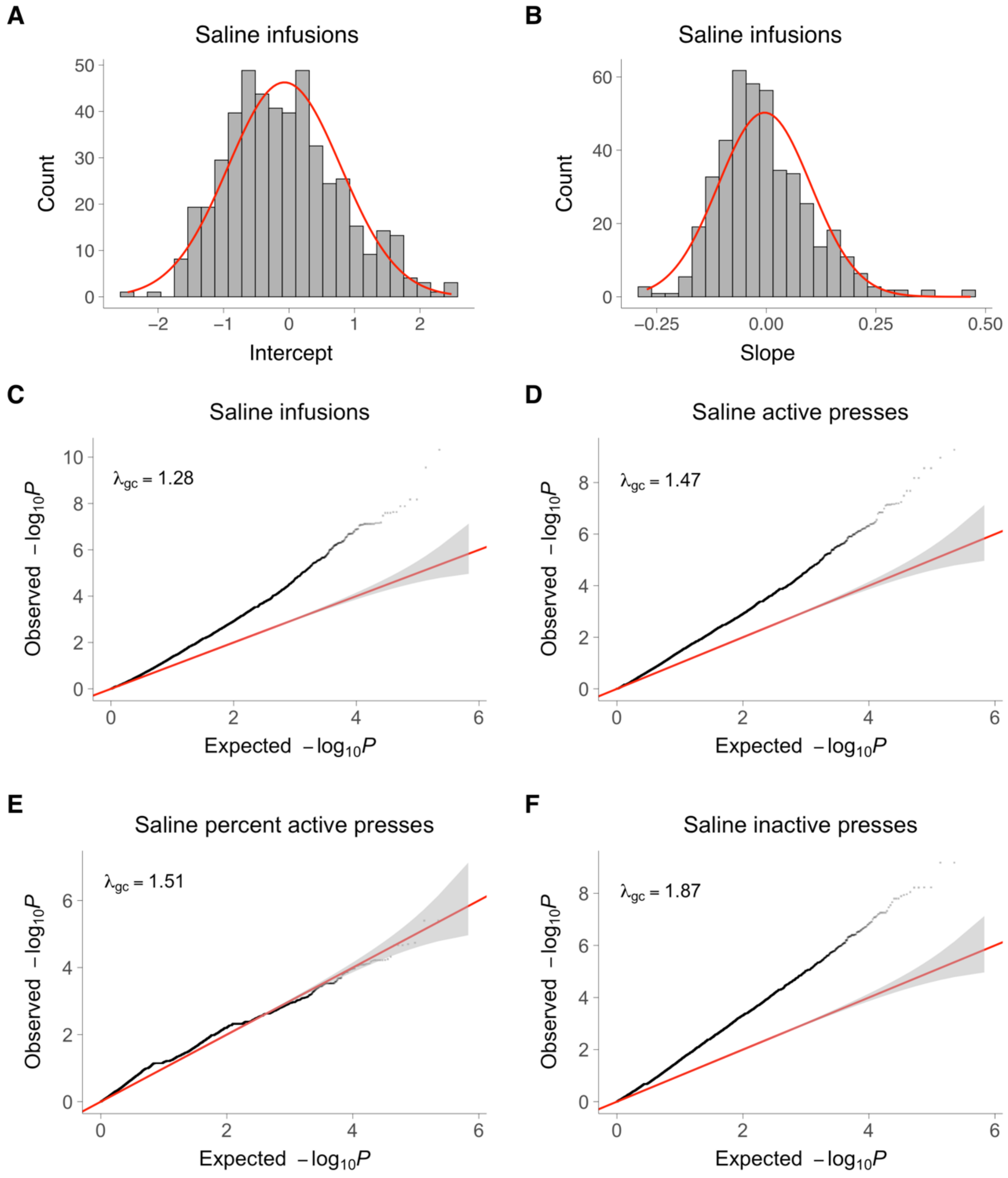

FIGURE S1 Intercepts, slopes and quantile-quantile (QQ) plots for longitudinal saline genome scans. (A) Infusion intercepts for individual mice (gray histogram bars). Normal distribution (red). (B) Infusion slopes for individual mice. (C) QQ plots for infusions. QQ plot for normal distribution (red). Grey area, 95% confidence interval. λ_{gc}, genomic control (inflation) factor. (D) Active lever presses. (E) Percent active lever presses. (F) Inactive lever presses.

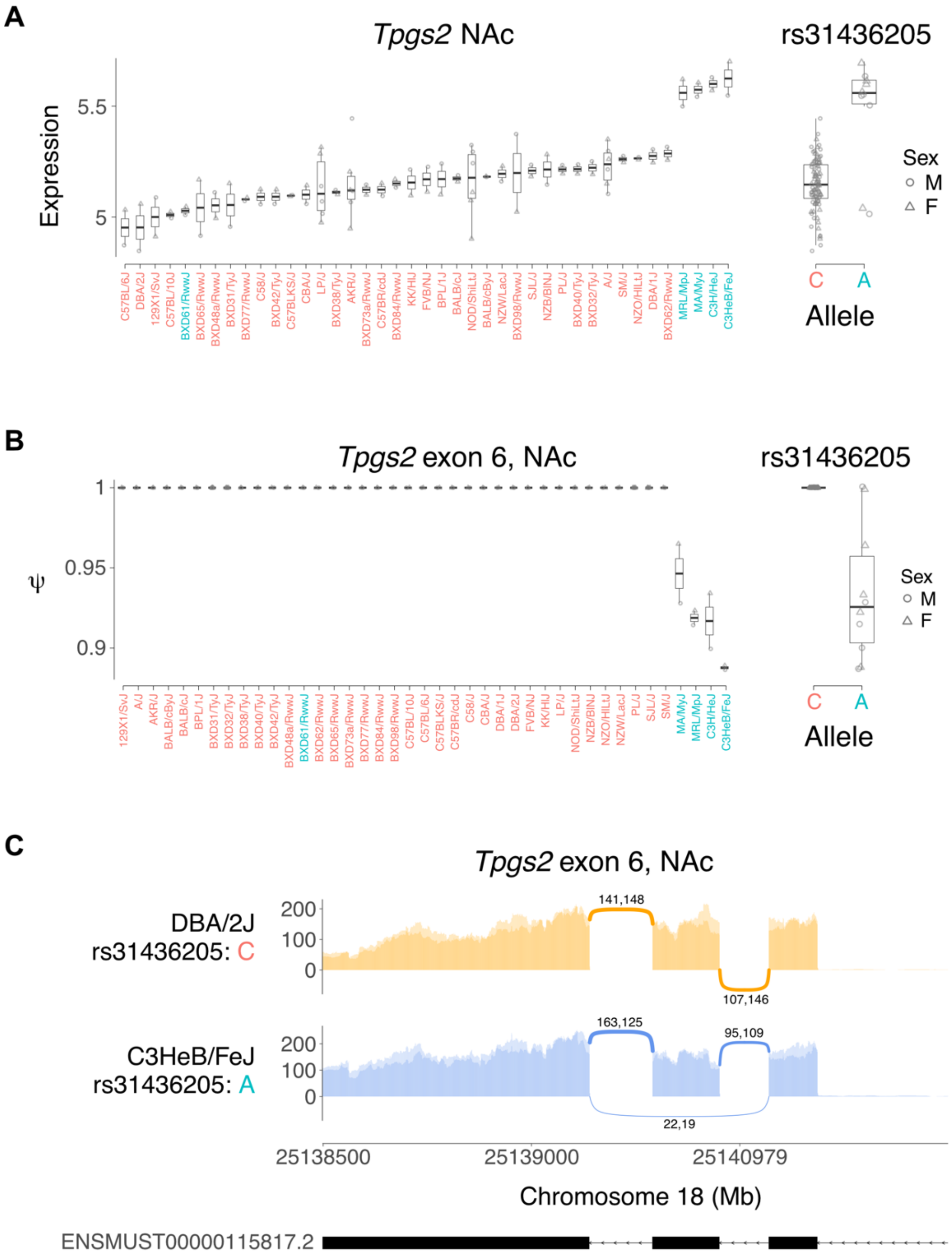

FIGURE S2 Genetic variation in alternate splicing of exon 6 *Tpgs2* in saline treated mice. (A) Strain survey of *Tpgs2* expression in saline NAc. Peak eQTL for *Tpgs2* is rs31436205. Strains with C allele tend to have low expression (red), while strains with A allele have high expression (blue). (B) Percent spliced in (psi or ψ) for exon 6 of *Tpgs2*. Strains with C allele have close to 100% ψ, while strains with A allele have lower ψ. (C) Sashimi plot [1] showing high ψ for *Tpgs2* exon 6 in DBA2/J, a strain with C allele of rs31436205, and low ψ for C3HeB/FeJ, a strain with A allele.

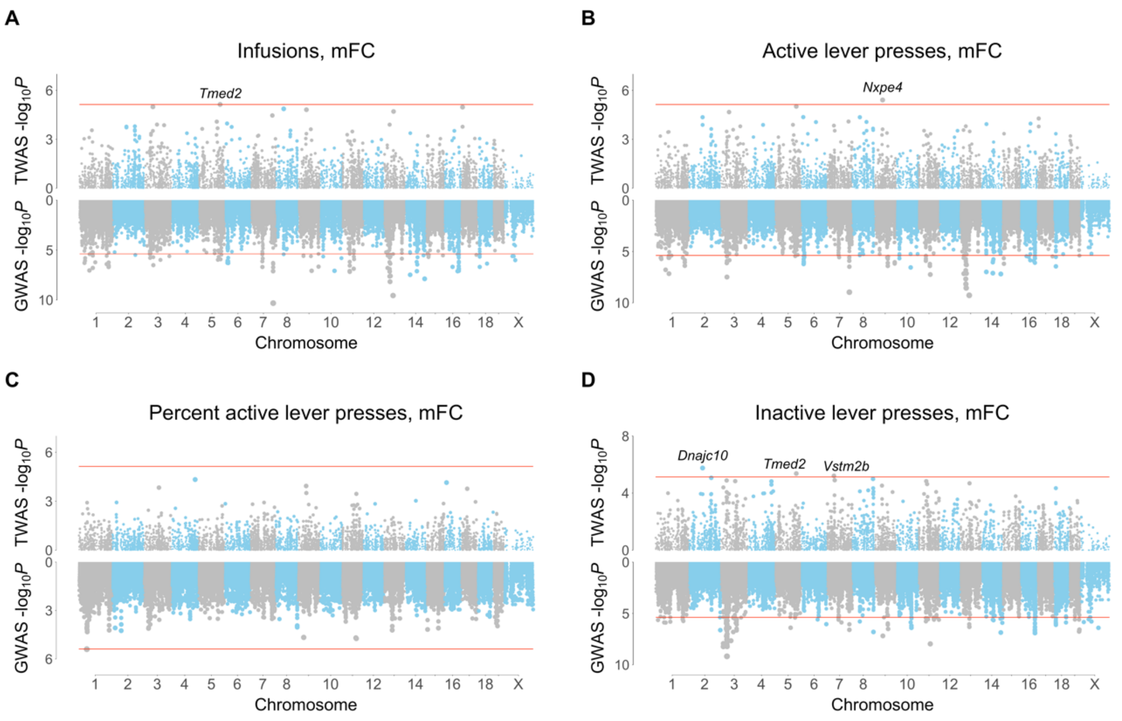

FIGURE S3 FUSION TWASs for saline IVSA using mFC RNA-Seq. (A) Infusions. (B) Active lever presses. (C) Percent active lever presses. (D) Inactive lever presses. TWASs on top, GWASs on bottom, with respective thresholds indicated by horizontal red lines.

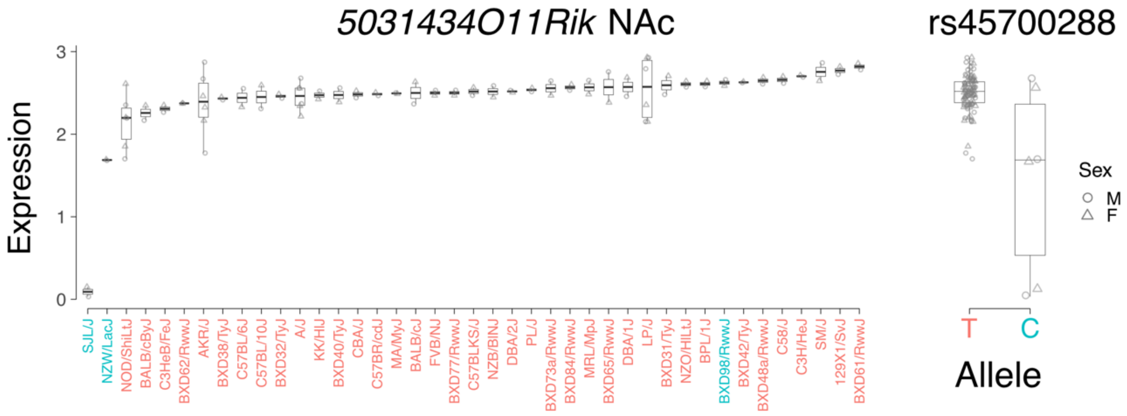

FIGURE S4 Expression of *5031434O11Rik* in saline treated mice. Strain survey of *5031434O11Rik* expression in NAc (left). Peak eQTL for *Tpgs2* is rs45700288 (right). Strains with T allele have high expression (red), while strains with the (minor) C allele have low expression (blue). Each datapoint for the C allele of rs45700288 (right) represents an average of pooled samples from 3 individuals (male or female).

REFERENCES

1. D. Garrido-Martín, E. Palumbo, R. Guigó, and A. Breschi, "ggsashimi: Sashimi Plot Revised for Browser- and Annotation-Independent Splicing Visualization," *PLOS Computational Biology* 14, no. 8 (2018): e1006360, https://doi.org/10.1371/journal.pcbi.1006360.
