# Supplementary material for: Differing genetics of saline and cocaine self-administration in the hybrid mouse diversity panel
Source: bioRxiv. 2024 Dec 9:2024.12.04.626933. Preprint. [Version 1] doi: 10.1101/2024.12.04.626933 (PMC11661131; doi:10.1101/2024.12.04.626933)
Supplement: Supplementary file 1 [file media-2.pdf]

Supplementary information:
Differing genetics of saline and cocaine self-administration in the hybrid mouse diversity panel

Arshad H. Khan[1,6], Jared R. Bagley[2,7], Nathan LaPierre[3,8], Carlos Gonzalez-Figueroa[4], Tadeo C. Spencer[4], Mudra Choudhury[4,9], Xinshu Xiao[4], Eleazar Eskin[5], James D. Jentsch[2], Desmond J. Smith[1,10]

[1] Department of Molecular and Medical Pharmacology, Geffen School of Medicine, UCLA, Los Angeles, CA 90095

[2] Department of Psychology, Binghamton University, Binghamton, NY 13902

[3] Department of Computer Science, UCLA, Los Angeles, CA 90095

[4] Department of Integrative Biology and Physiology, UCLA, Los Angeles, CA 90095

[5] Department of Computational Medicine, UCLA, Los Angeles, CA 90095

[6] Current address: Cedars-Sinai Medical Center, 8700 Beverly Blvd, Los Angeles, CA 90048

[7] Current address: Department of Pharmaceutical Sciences, Binghamton University, Binghamton, NY 13902

[8] Current address: Department of Human Genetics, University of Chicago, Chicago, IL 60637

[9] Current address: Sanford Burnham Prebys, La Jolla, CA 92037

[10] To whom correspondence should be addressed:
Department of Molecular and Medical Pharmacology
David Geffen School of Medicine, UCLA
Box 951735, 23-151A CHS
Los Angeles, CA 90095-1735
Email: DSmith@mednet.ucla.edu

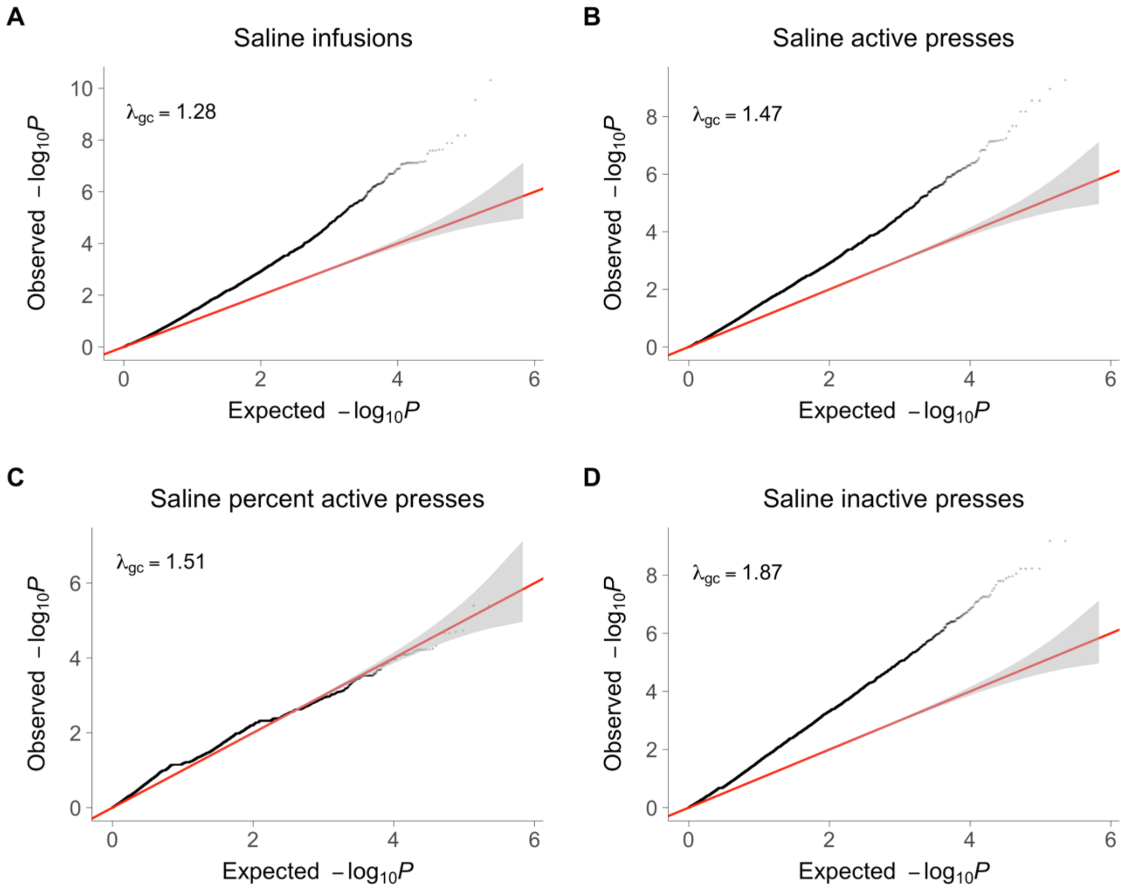

Figure S1. *Quantile-quantile (QQ) plots for longitudinal saline genome scans. (**A**) Infusions. (**B**) Active lever presses. (**C**) Percent active lever presses. (**D**) Inactive lever presses.* λ_{gc}*, genomic control (inflation) factor.*

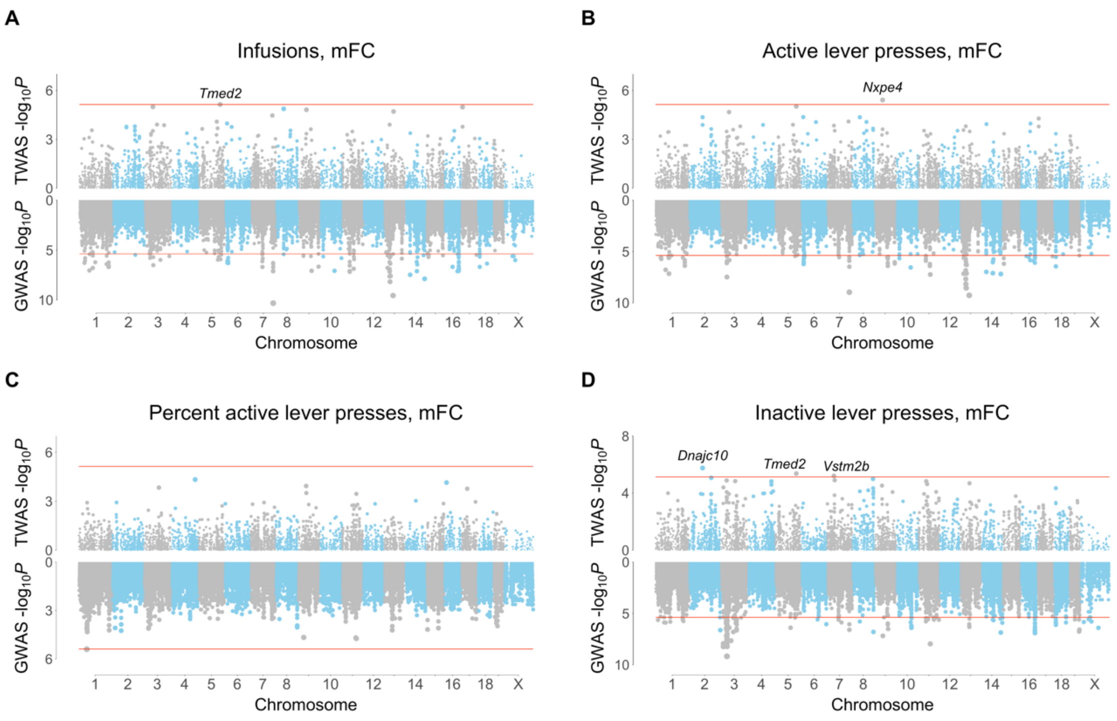

Figure S2. *`FUSION` TWAS for saline IVSA using mFC RNA-Seq. (**A**) Infusions. (**B**) Active lever presses. (**C**) Percent active lever presses. (**D**) Inactive lever presses. TWASs on top, GWASs on bottom.*
